# Veterinary Department, Columbian University

**Published:** 1897-09

**Authors:** 


### VETERINARY DEPARTMENT, COLUMBIAN UNIVERSITY.

The Hon. Edwin Willits has been dropped from the Faculty and Drs. Victor A. Norgaard and David McMaster added; also Dr. William H. Bolyn. Dr. Glass's translation of Dr. Müller's *Diseases of the Dog* has been added to the list of text-books.

